# Enzymes involved in vinyl acetate decomposition by *Pseudomonas fluorescens* PCM 2123 strain

**DOI:** 10.1007/s12223-013-0268-0

**Published:** 2013-08-03

**Authors:** Elżbieta Szczyrba, Izabela Greń, Grażyna Bartelmus

**Affiliations:** 1 Institute of Chemical Engineering, Polish Academy of Sciences, Bałtycka 5, 44-100 Gliwice, Poland; 2 Department of Biochemistry, Faculty of Biology and Environment Protection, University of Silesia, Jagiellońska 28, 40-032 Katowice, Poland; 3 Department of Process Engineering, University of Opole, Dmowskiego 7-9, 45-365 Opole, Poland

## Abstract

Esterases are widely used in food processing industry, but there is little information concerning enzymes involved in decompositions of esters contributing to pollution of environment. Vinyl acetate (an ester of vinyl alcohol and acetic acid) is a representative of volatile organic compounds (VOCs) in decomposition, of which hydrolyses and oxidoreductases are mainly involved. Their activities under periodically changing conditions of environment are essential for the removal of dangerous VOCs. Esterase and alcohol/aldehyde dehydrogenase activities were determined in crude cell extract from *Pseudomonas fluorescens* PMC 2123 after vinyl acetate induction. All examined enzymes exhibit their highest activity at 30–35 °C and pH 7.0–7.5. Esterase preferably hydrolyzed ester bonds with short fatty chains without plain differences for C_2_ or C_4_. Comparison of Km values for alcohol and aldehyde dehydrogenases for acetaldehyde suggested that this metabolite was preferentially oxidized than reduced. Activity of alcohol dehydrogenase reducing acetaldehyde to ethanol suggested that one mechanism of defense against the elevated concentration of toxic acetaldehyde could be its temporary reduction to ethanol. Esterase activity was inhibited by phenylmethanesulfonyl fluoride, while β-mercaptoethanol, dithiothreitol, and ethylenediaminetetraacetic acid had no inhibitor effect. From among metal ions, only Mg^2+^ and Fe^2+^ stimulated the cleavage of ester bond.

**Electronic supplementary material:**

The online version of this article (doi:10.1007/s12223-013-0268-0) contains supplementary material, which is available to authorized users.

## Introduction

Vinyl acetate belongs to the group of volatile organic compounds (VOCs). In the industry, it is used for the production of polyvinyl acetate, polyvinyl alcohol, and other polymers which are finally exploited to manufacture building materials, printing inks, plastics, lacquers, or paints (US EPA 2000). Simultaneously, vinyl acetate appears in exhaust gases, and although it itself is not considered to be highly toxic, it is very hazardous in case of skin and eye contact as well as in case of inhalation (ATSDR 1992). When released into the environment (mainly into air), it can be broken down by photochemical pathways involving hydroxyl radicals or ozone (Ferreira et al. 2010; Kaczmarek et al. 2002). Products of its degradation such as, e.g., acetaldehyde are thought to be more toxic (Greń et al. 2009; Nieder et al. 1990).

So far, only few papers focused on vinyl acetate decomposition by microorganisms (Greń et al. 2009; Greń et al. 2011; Hatanaka et al. 1989; Kasperczyk et al. 2007; Lara-Mayorga et al. 2010; Nieder et al. 1990). Ability to metabolize vinyl acetate depends on the capacity for synthesis of carboxyl esterase (EC 3.1.1.1). There have been published many reports characterizing esterases employed in food processing industry (Castillo et al. 1999; Choi and Lee 2001; Choi et al. 2004; Fenster et al. 2003a, b; Gobbetti et al. 1997a, b; Liu et al. 2001). However, there is a shortage of data concerning properties of vinyl acetate esterase used in waste gases biotreatment technology. Characterization of carboxylesterase from vinyl acetate-assimilating bacterium has been so far described only by Hatanaka et al. (1989). They described three types of esterases in *Pseudomonas* sp. Z2 strain from which only one was involved in the assimilation of vinyl acetate. Apart from the determination of esterase substrate specificity, there were no other studies concerning the optimization of environmental conditions for its activity.

**Fig. 1:**
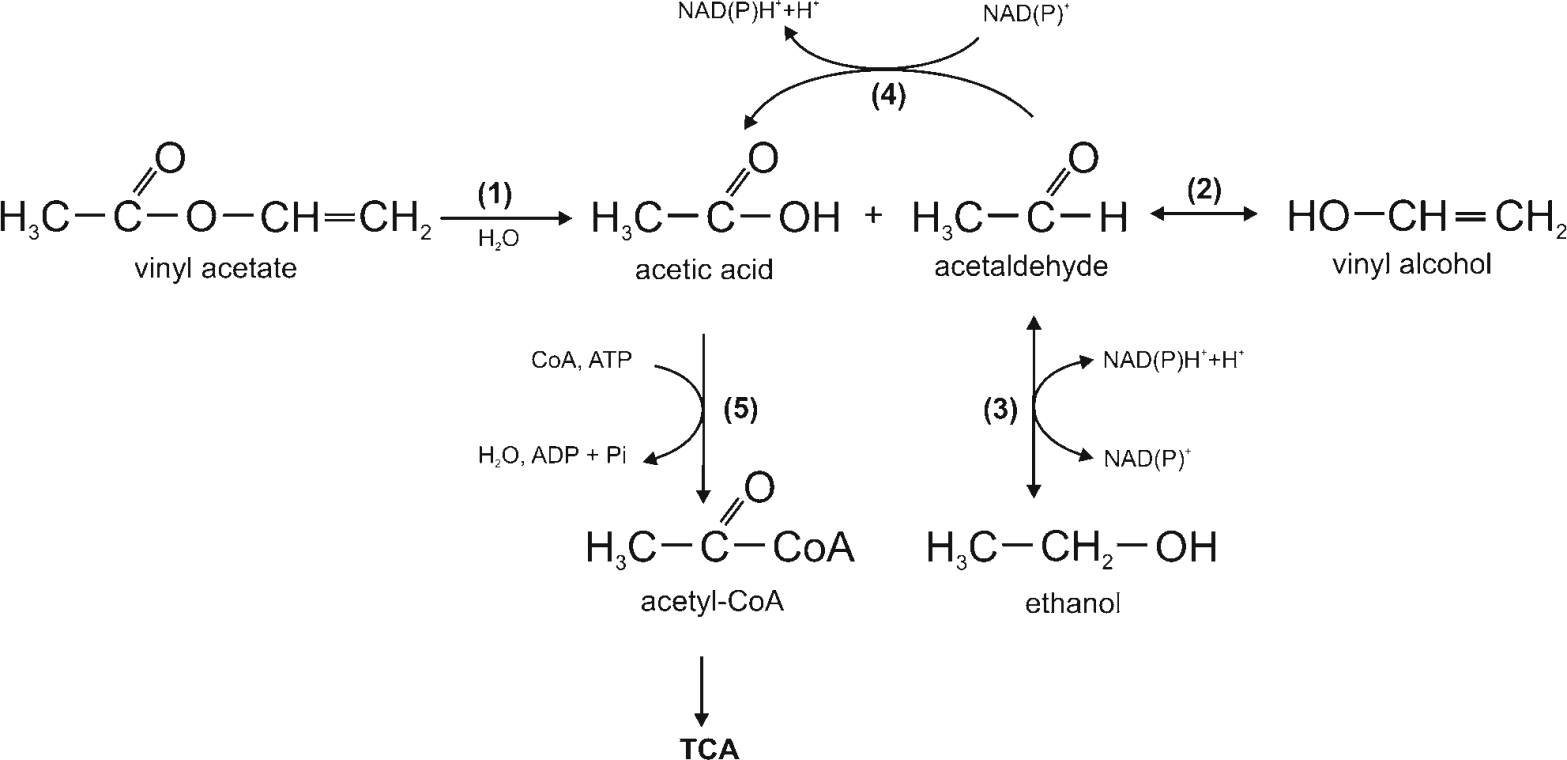
Proposed pathway of vinyl acetate degradation in *P*. *fluorescens* PCM 2123 cells, where: (*1*) carboxylesterase, (*2*) tautomerization (chemical process), (*3*) alcohol dehydrogenase, (*4*) aldehyde dehydrogenase, (*5*) acetylo-CoA synthetase, *TCA* tricarboxylic acid cycle (Nieder et al. 1990, modified)

Esterases are described as enzymes catalyzing both cleavage and formation of ester bonds. They hydrolyze the fatty acid esters with short chains and prefer water-soluble substrates like vinyl acetate (Ateslier and Metin 2006). Products of vinyl acetate cleaving by esterase are thought to be further degraded as shown in Fig 1. Because of the toxicity of acetaldehyde, some microorganisms create very simple and efficient mechanism of provisional decline of its concentration. One part of acetaldehyde is directly metabolized, while the second one is temporarily converted into ethanol, which is afterwards metabolized in the opposite reaction to acetaldehyde. Both reactions are catalyzed by the same oxidoreductase (EC 1.1.1.-): alcohol dehydrogenase cooperating with NADH while it reduces acetaldehyde to its alcohol form, or NAD^+^ while it oxidizes ethanol (Fig. 1) (Nieder et al. 1990).

Previously, we described the susceptibility profile of *Pseudomonas fluorescens* PCM 2123 to vinyl acetate (Greń et al. 2009), as well as its ability to vinyl acetate decomposition (Greń et al. 2011). Kinetics of vinyl acetate biodegradation by PCM 2123 strain and its modeling on the laboratory scale has been studied (Gąszczak et al. 2010; Kasperczyk et al. 2007; Kasperczyk and Bartelmus 2010). PCM 2123 strain will be used to construct the trickle-bed bioreactor operating at production scale to purify the outlet gases, and therefore it must be able to perform vinyl acetate decomposition under changeable environmental conditions. The aim of these studies was to determine the optimal conditions for activities of esterase as the essential enzyme in degradation of vinyl acetate by *P*. *fluorescens* PCM 2123. Activities of dehydrogenases involved in subsequent metabolism of products of ester bond cleavage were also verified.

## Materials and methods

### Bacterial strain, growth conditions, and preparation of crude enzyme extract

*P*. *fluorescens* PCM 2123 was obtained from The Polish Collection of Microorganisms (Wrocław). Bacteria were grown in mineral medium, and its composition was described previously (Greń et al. 2009). Vinyl acetate in concentration of 0.4 g/L was added to the culture every 24 h as a source of carbon and energy. The strain was grown aerobically for 5 to 7 days in a shaker at 130 rpm at 30 °C in 500 mL Erlenmeyer flasks. Cells were harvested in the late exponential phase as performed in Greń et al. (2011). The protein concentration was estimated using the Bradford method with lysozyme as a standard (Bradford 1976).

### Enzyme assays

Esterase activity was determined spectrophotometrically using *p*-nitrophenyl butyrate (*p*NPB) or *p*-nitrophenyl acetate (*p*NPA) as described previously (Greń et al. 2011). Dehydrogenase activities were determined spectrophotometrically, as it was presented earlier (Greń et al. 2011). Each measurement was performed in triplicates. One unit (U) of esterase activity was defined as the amount of enzyme releasing 1 μmol of *p*-nitrophenol per minute under assay conditions.

### Effect of temperature on enzyme activity

The temperature dependence on the esterase activity was determined in 50 mmol/L phosphate buffer, pH 7.5, in the range 5–50 °C at every 5 °C. Buffer was preincubated at the examined temperature, and reaction was started by substrate addition.

Dehydrogenase activity was examined in the same buffer at various temperatures from 5 to 40 °C at every 5 °C.

### Thermostability

The enzyme was preincubated in temperatures between 5 and 65 °C at every 5 °C for 30 min. After incubation, samples were cooled on ice, centrifuged, and the remaining activity was measured with *p*NPB as a substrate. As a control (100 %), the initial activity of enzyme measured in standard conditions was used.

### Determination of pH optimum

The effect of pH on enzymes activity was determined by measuring activity at 30 °C over pH range of 4.0–8.0 using 0.05 mol/L phosphate–citrate (pH 3.0–5.0) and 0.05 mol/L Sörensen (pH 6.0–8.0) buffer.

**Table 1:** Kinetics parameters of enzymes activities involved in vinyl acetate decomposition

Enzyme	Substrate	Vmax (mU/mg protein)	Km (mmol/L)
Esterase			
	*p*NPA	150	0.045
	*p*NPB	116	0.058
Alcohol dehydrogenase			
	Ethanol	68	0.022
	Ethanal	112	0.3
Aldehyde dehydrogenase			
	Ethanal	286	0.057

### pH stability

Crude enzyme extract was preincubated for 30 min at 30 °C in buffers of the different pH in the range of 3.0–8.0. After incubation, samples were cooled on ice, centrifuged, and the residual activity was measured using *p*NPB. As a control (100 %), the initial activity of enzyme measured in standard conditions was used.

### Enzyme kinetics

Measurements were performed under standard conditions using various concentrations from 0.025 to 2 mmol/L of *p*NPA or *p*NPB. The crude cell extract was incubated with various concentration of acetaldehyde for aldehyde/alcohol dehydrogenase to give final substrate concentrations in the range of 0.01 to 0.5 mmol/L or ethanol for alcohol dehydrogenase in the range of 0.01 to 2.5 mmol/L.

Michaelis–Menten constant (Km) and the maximum velocity of the reactions were calculated from a Lineweaver–Burk plot (Table 1).

### Substrate specificity

Different esters: *p*NPB, *p*NPA (in DMSO), or *p*-nitrophenyl palmitate (*p*NPP) (in acetone) were tested for esterase activity. The measurements were performed under standard assay conditions.

### Effect of metal ions

The crude enzyme extract was preincubated for 30 min at 30 °C with 1 mmol/L various metal ions solutions (Table 2). Residual esterase activity was measured under standard conditions with *p*NPB as a substrate. As a control (100 %), activity of enzyme after incubation without any metal ion was used.

**Table 2:** Effect of metal ions (concentration 1 mmol/L) on the activity of vinyl acetate esterase

Metal ion (salts)	Residual activity (%)
None	100
Zn^2+^_1_	87
Mg^2+^_2_	121
Al^3+^_1_	89
Ca^2+^_1_	96
Cd^2+^_1_	99
Mn^2+^_3_	89
Hg^2+^_1_	87
Ni^2+^_1_	95
Fe^2+^_2_	180

*1* added as chloride

*2* added as monohydrate chloride

*3* added as monohydrate sulfate (VI)

### Effect of inhibitors

The crude enzyme extract was preincubated for 30 min at 30 °C in 1 mmol/L solutions of different inhibitors (Table 3), apart from phenylmethanesulfonyl fluoride (PMSF) that was prepared in three different concentrations: 0.1, 1, and 5 mmol/L. The remaining activity was determined under standard conditions with *p*NPB as a substrate. As a control, (100 %) activity of enzyme after incubation without any potential inhibitor was used.

**Table 3:** Effect of inhibitors on the activity of vinyl acetate esterase

Chemicals	Concentration(mmol/L)	Relative activity (%)
None	–	100
PMSF	0.1	38
PMSF	1	16
PMSF	5	0
β-ME	1	144
Tween 80	1	159
DTT	1	141
Triton X-100	1	114
EDTA	1	159

### Chemicals

Analytical reagent grade chemicals were obtained from Sigma (St Louis, MO, USA), Merck (Darmstadt, Germany), Fluka (Buchs, Switzerland), or POCh (Gliwice, Poland).

## Results and discussion

### Effect of temperature on enzyme activity and stability

**Fig. 2:**
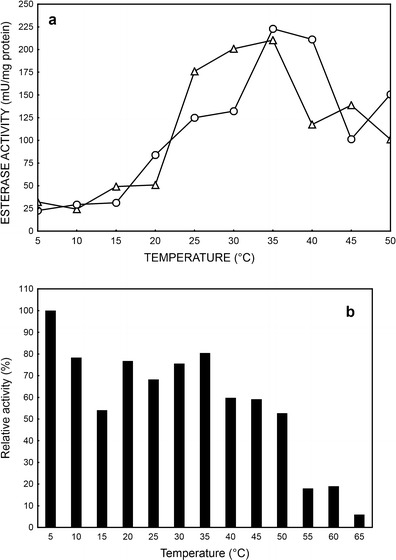
Effect of temperature (**a**) and temperature stabilization (**b**) on esterase activity from PCM 2123 cells, where as a substrate in reaction mixture was used: *p*NPB (*open triangle*) or *p*NPA (*open circle*)

The esterase showed maximum activity at temperature 35 °C nevertheless of substrate used in reaction mixture (Table 4, Supplement). When temperature dropped to about 20 °C, esterase exhibited 20–35 % of its maximal activity (Fig. 2a). Similar results were obtained by Castillo et al. (1999) for *Lactobacillus casei* subsp. *casei* IFLP31 with optimal temperature at 25–30 °C and Gobbetti et al. 1997b for *Lactobacillus fermentum* DT41 with optimal temperatures of esterase activity at 30–35 °C. On the other hand, Ateslier and Metin (2006) described strain *Bacillus* sp. 4 with optimal growth and esterase production at 65 °C. Enzymes exhibiting stability in high temperatures are supposed to be more proper for harsh conditions of industrial processes, but on the other hand, their activities are relatively low at room temperature, which is sometimes required for chemicals labile in higher temperature like, e.g., vinyl acetate (Zamost et al. 1991).

**Fig. 3:**
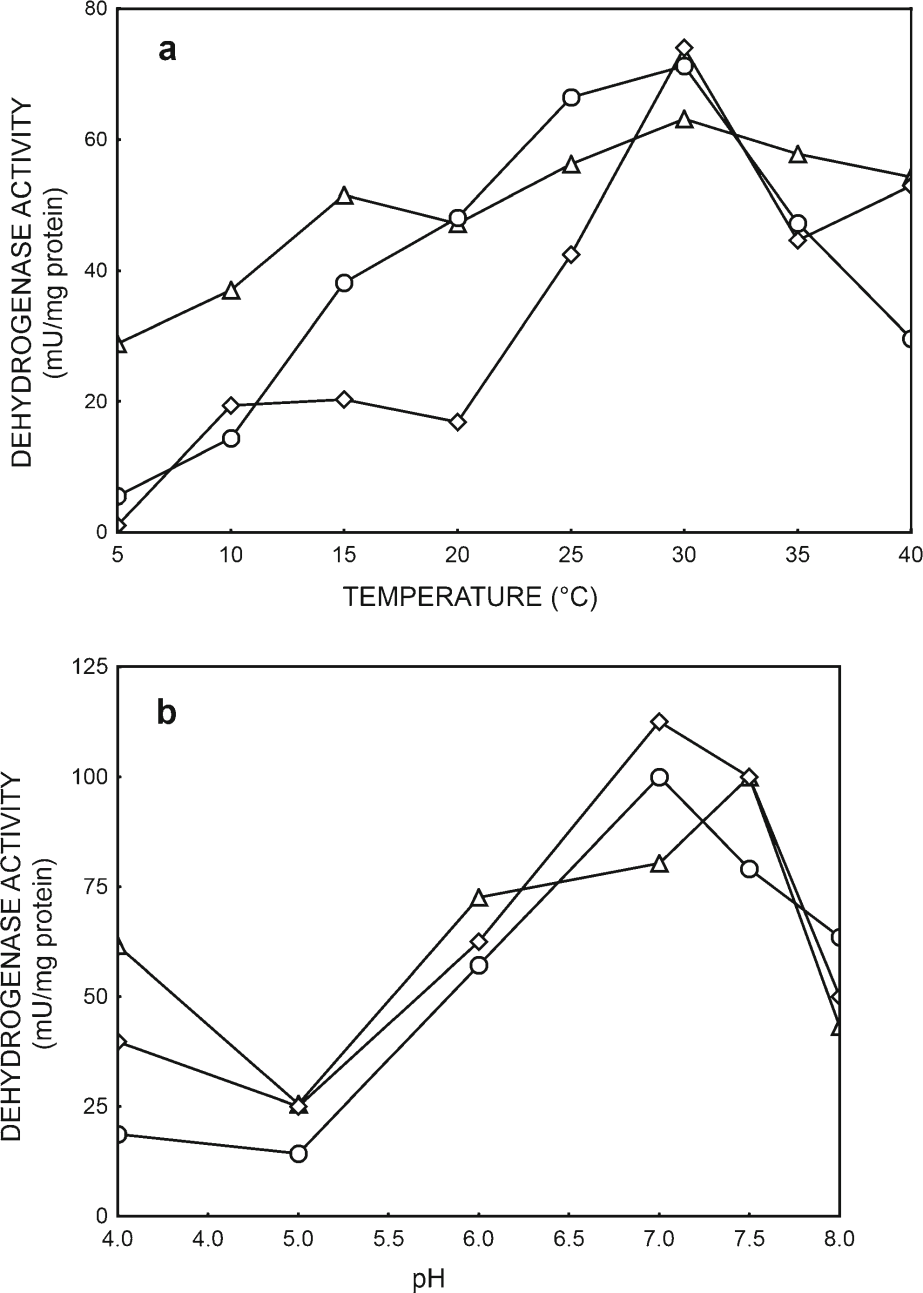
Effect of temperature (**a**) and pH (**b**) on specific activities of dehydrogenase from PCM 2123 cells, where: alcohol dehydrogenase (ethanol) (*open triangle*), alcohol dehydrogenase (acetaldehyde) (*open circle*), aldehyde dehydrogenase (*open diamond*)

The optima temperatures for activities of dehydrogenases from PCM 2123 cells were at 30 °C (Fig. 3a), and an aldehyde dehydrogenase was the only one which lost almost 80 % of its activity when temperature decreased to 20 °C.

**Fig. 4:**
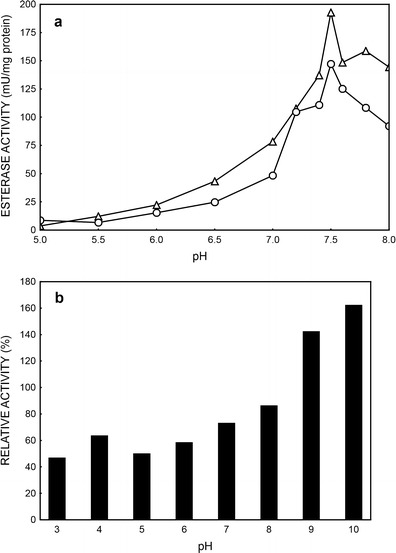
Effect of pH (**a**) and pH stabilization (**b**) on esterase specific activity from PCM 2123 cells, where as a substrate in reaction mixture was used: *p*NPB (*open triangle*) or *p*NPA (*open* c*ircle*)

The results referring to thermostability of esterase from PCM 2123 cells showed that enzyme is stable (relative activity higher near 80 %) at temperature from 5 to 35 °C, and above 50 °C, its activity decreases sharply (Fig. 2b). The similar method of testing esterase thermostability was used by Sana et al. (2007), but preincubation time was prolonged to 1 h. Esterase from a salt-tolerant *Bacillus* sp. strain BSE01 showed no difference in activity up to 50 °C, and it is more stable at higher temperature than the esterase isolated from strain PCM 2123. Thermostable enzymes like esterase described by Sobek and Görisch (1988) from thermophilic archaebacterium *Sulfolobus acidocaldarius* lost only 8 % of its activity at 90 °C and 50 % at 100 °C.

### Determination of pH optima and pH stability

The pH optimum of esterase (Fig. 4a) was about pH 7.5, and dehydrogenases showed the highest activities also in the pH close to neutral (Fig. 3b). When pH of environment was increased to 10, the activity of esterase dramatically decreased (data not shown). The esterase shows a slightly higher activity in the presence of *p*NPB than *p*NPA at different pH (Fig. 4a). The relative activity of the enzyme was higher than 70 % at pH from 7.4 to 8 for *p*NPB and at pH from 7.2 to 7.8 for *p*NPA. Similar results were observed for esterases isolated from numerous microorganisms, e.g., *L*. *casei* subsp. *casei* IFLP31, with pH optimum about 7.5 (Castillo et al. 1999) or from *Bacillus* species—optimal pH about 6.0 (Ateslier and Metin 2006). It is thought that esterases show the highest activity at neutral pH, and this property distinguish them from lipases, which are most active at pH above 8 (Ateslier and Metin 2006).

The first step of testing pH stability of esterase from PCM 2123 cells was preincubation of crude cell extract in buffers of different pH (Fig. 4b). The activity of esterase in the pH range from 3.0 to 7.0 was relatively stable, although it increased when pH rose above 8, which was described for thermophilic *Bacillus* sp. by Ateslier and Metin (2006). They suggest that the highest activity at pH 9 or 10 could be connected with the presence of some proteases in the crude enzyme extracts. These proteases probably exhibit high activity at pH 7.5 and digest the esterase causing decrease of its activity. While the pH value of environment reaction rises, the activity of proteases is reduced.

### Enzyme kinetics

The kinetic parameters of vinyl acetate esterase like the Michaelis–Menten constant (Km) and velocity for the reaction (V_max_) were estimated to be, respectively, 0.045 mmol/L and 150 mU/mg protein for *p*NPA and 0.058 mmol/L and 116 mU/mg protein for *p*NPB (Table 1). Shortage of data concerning kinetics of vinyl acetate by other microorganisms makes some difficulties to compare these data with the others. The Km value seems to be similar to the other esterases, e.g., from *Bacillus* sp. 4, it was 62.89 μmol/L (Ateslier and Metin 2006) or from *L*. *casei* LILA, it was 97 μmol/L (Fenster et al. 2003a), when *p*-nitrophenyl butyrate as a substrate were used. It has been previously shown that for numerous industrially relevant enzymes, Km values are in the range of 10^−1^ to 10^−5^ mol/L (Fullbrook 1996). Maximal velocities (V_max_) of reactions catalyzed by esterases are of different values. Some of them exceeded the results for vinyl acetate esterase from PCM 2123 strain, e.g., V_max_ of esterase isolated from *Bacillus* sp. 4 was 833.33 mU/mg protein, when *p*NPB was used as a substrate (Ateslier and Metin 2006), or they are of the same value or lower (Castillo et al. 1999; Fenster et al. 2003a; Yang and Liu 2008). Km and V_max_ values for both oxidoreductases involved in the metabolism of vinyl acetate products were also determined (Table 1). The highest V_max_ for alcohol dehydrogenase was determined, when acetaldehyde was used as a substrate in the reaction mixture. Comparison of Km values for alcohol and aldehyde dehydrogenases for acetaldehyde suggests that this metabolite of vinyl acetate decomposition will be preferentially oxidized than reduced. Activity of alcohol dehydrogenase reducing acetaldehyde to ethanol confirms our suggestions that one mechanism of defense against elevated concentrations of toxic acetaldehyde can be its temporary reduction to ethanol.

### Substrate specificity

In order to estimate the substrate specificity of esterase, the following chemicals: *p*NPA (C_2_), *p*NPB (C_4_), or *p*NPP (C_16_) were added to the reaction mixture. It was observed that esterase was active for short-chain fatty acid substrates like *p*NPB (192.77 ± 42.18 mU/mg protein) and *p*NPA (147.15 ± 31.11 mU/mg protein), and it completely lost its activity for *p*NPP. Statistical analysis did not show any significant difference for vinyl acetate esterase with *p*NPB or *p*NPA as a substrate. These results are similar to that obtained for the other described esterases (Ateslier and Metin 2006; Fenster et al. 2003a; Gobbetti et al. 1997a; Kim et al. 2006; Liu et al. 2001; Sobek and Görisch 1988), and are characteristic for hydrolyzes belonging to esterases usually hydrolyzing esters with short-chain fatty acid (C_2_ to C_4_). Only recombinant esterase from *L*. *casei* CL96 described by Choi and Lee (2001) showed the major activity for fatty acid esters containing C_6_ and C_8_.

### Effect of effectors on esterase activity

Assays to determine the effects of metal ions on esterase activity, using *p*NPB as a substrate, showed that various metal ions influence in different way on the activity of vinyl acetate esterase (Table 2). Mg^2+^ and Fe^2+^ were found to stimulate its activity. Esterase from *L*. *fermentum* DT41 also revealed higher activity in the presence of Mg^2+^, but Fe^2+^ ion inhibited esterase activity (Gobbetti et al. 1997b). There was no influence of Ni^2+^, Ca^2+^, Cd^2+^ ions on vinyl acetate esterase from PCM 2123 cells; however, Ca^2+^ was found to slightly enhance (Choi et al. 2004; Gobbetti et al. 1997b; Tekedar and Sanli-Mohamed 2011) or inhibit (Castillo et al. 1999) activity of this type of enzyme. Other examined ions like Zn^2+^, Al^3+^, Mn^2+^, and Hg^2+^ slightly inhibited the esterase, although the Hg^2+^ ion is usually the strong inhibitor of esterases because of the reaction with thiol groups (Choi et al. 2004; Gobbetti et al. 1997b).

The vinyl acetate esterase showed higher activity in the presence of reducing agent like DTT, β-mercaptoethanol (β-ME), and EDTA. These results are similar to those obtained by Tekedar and Sanli-Mohamed (2011) for esterases isolated from three different *Geobacillus* strains. However, these chemicals usually cause decrease of enzyme activity or even total inhibition. The β-ME reacts with sulfhydryl groups and disulfide bonds existing in catalytic side, and thanks to them, the active conformation of enzyme could be maintained, and the presence of β-ME in the reaction mixture causes decrease of its activity (Choi et al. 2004; Yang and Liu 2008). Our data suggest that thiol groups were not present or were not essential for the catalytic activity if vinyl acetate esterase from *P*. *fluorescens*.

The examined esterase was resistant to some detergents like Triton X-100 and showed typical for esterases property to be strongly inhibited by PMSF known as a serine inhibitor (Table 3). This is connected with the presence a serine residue in catalytic side, what could be observed equally in lipases and esterases, but lipase possess a characteristic lid, which cover the active side, and makes it resistant to PMSF. The esterases do not have this hydrophobic domain, so they are strongly inhibited by PMSF even in low concentration (Ateslier and Metin 2006; Castillo et al. 1999; Gobbetti et al. 1997a, b; Sana et al. 2007; Smacchi et al. 2000).

These results suggest that the examined enzymes of vinyl acetate biodegradation pathway in PCM 2123 cells, and especially vinyl acetate esterase, exhibit catalytic activities under changeable conditions. Determination of optimal conditions for enzymes activities designates *P*. *fluorescens* PCM 2123 for some biotechnological application, especially for vinyl acetate removing from exhausted gases, even when the harsh conditions will appear.

## Electronic supplementary material

Below is the link to the electronic supplementary material.

ESM 1 (DOCX 20 kb)
