# Supplementary material for: Enzymes involved in vinyl acetate decomposition by Pseudomonas fluorescens PCM 2123 strain
Source: Folia Microbiol (Praha). 2013 Aug 3;59(2):99–105. doi: 10.1007/s12223-013-0268-0 (PMC3936133; doi:10.1007/s12223-013-0268-0)
Supplement: Supplementary file 1 — (DOCX 20 kb) [file 12223_2013_268_MOESM1_ESM.docx]

Table 4

Esterase activity for *p*NPA or *p*NPB in different temperatures;

| Temperature, °C | *p*NPB | *p*NPA |
| --- | --- | --- |
| 5 | 32.14 ± 5.21a | 22.83 ± 3.73a |
| 10 | 24.23 ± 5.58a | 29.30 ± 2.69a |
| 15 | 49.17 ± 15.35a | 31.45 ± 10.45a |
| 20 | 50.93 ± 16.13a | 84.01 ± 19.04a |
| 25 | 176.08 ± 36.55a | 127.94 ± 33.21a |
| 30 | 200.86 ± 9.85a | 132.27 ± 18.39b |
| 35 | 210.39 ± 8.00a | 222.99 ± 0.00a |
| 40 | 124.56 ± 17.75a | 211.28 ± 40.41b |
| 45 | 138.91 ± 8.97a | 101.43 ± 43.94a |
| 50 | 101.25 ± 27.96a | 150.77 ± 33.39a |

Analyses of variance (ANOVA) were performed on at least three replicates data obtained; When results of ANOVA test were statistically significant (*p*< 0.05) they were assessed by post hoc comparison of means using lowest significant differences (LSD) test. All analyses were performed using the programme Statistica 10.0 PL. Means with the various letters were significantly different. Lack of letters denoted no significant difference.
